# Supplementary material for: Capillary Wicking on Heliamphora minor-Mimicking Mesoscopic Trichomes Array
Source: Biomimetics (Basel). 2024 Feb 9;9(2):102. doi: 10.3390/biomimetics9020102 (PMC10887133; doi:10.3390/biomimetics9020102)
Supplement: Supplementary file 1 [file biomimetics-09-00102-s001.zip › biomimetics-2833126-supplementary.pdf]

Supporting Information

Capillary wicking on *Heliamphora minor* mimetic mesoscopic trichomes array

Fenglin Chen [1, 2], *Ziyang Cheng* [1, 2], *Lei Jiang* [1, 2] *and Zhichao Dong* [1, 2]*

[1] CAS Key Laboratory of Bio-Inspired Materials and Interfacial Science, Technical Institute of Physics and Chemistry, Chinese Academy of Sciences, Beijing 100190, China

[2] School of Future Technology, University of Chinese Academy of Sciences, Beijing 100049, China

* Correspondence: dongzhichao@mail.ipc.ac.cn

Contents

Figure S1 Capillary wicking phenomena on trichomes arrays modified surface of different contact angles.
Figure S2 Dynamics of traditional capillary rise in a closed tube.

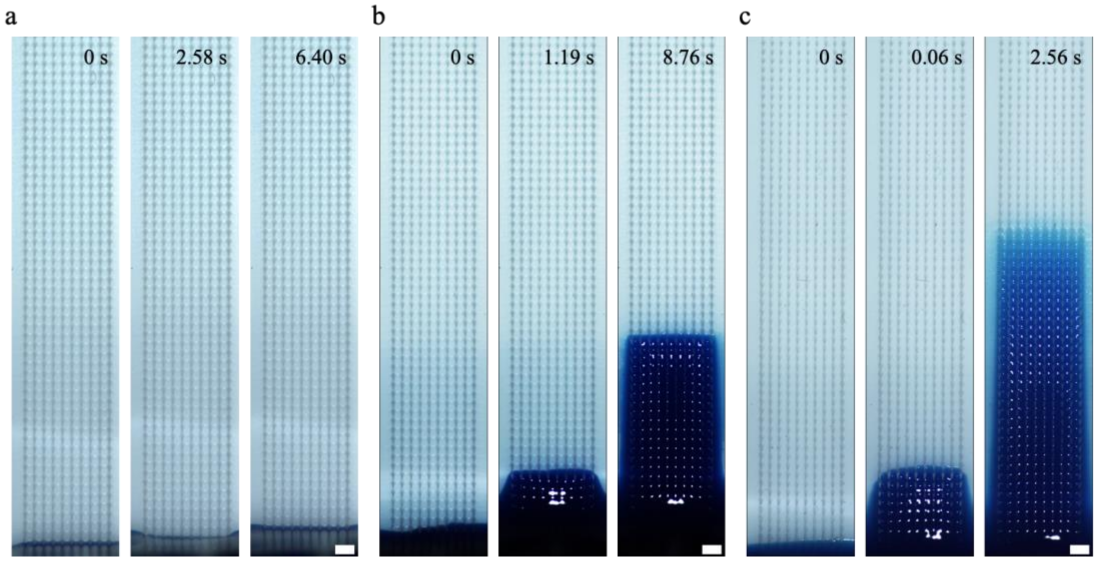

Figure S1 Capillary wicking phenomena on trichomes arrays modified surface of different contact angles. (**a**) Capillary wicking on hydrophobic surface whose contact angle is 117.2°, water does not wick on trichomes array even after 6.40 s; (**b**) Capillary wicking on hydrophilic surface whose contact angle is 63.8°, water wicks slowly on the substrate and reaches a height of 11.5 cm; (**c**) Capillary wicking on superhydrophilic surface whose contact angle is close to 0°, water wicks fast on the substrate and reaches a height of 18.0 cm. Scale bars, 1 mm in (**a**)-(**c**).

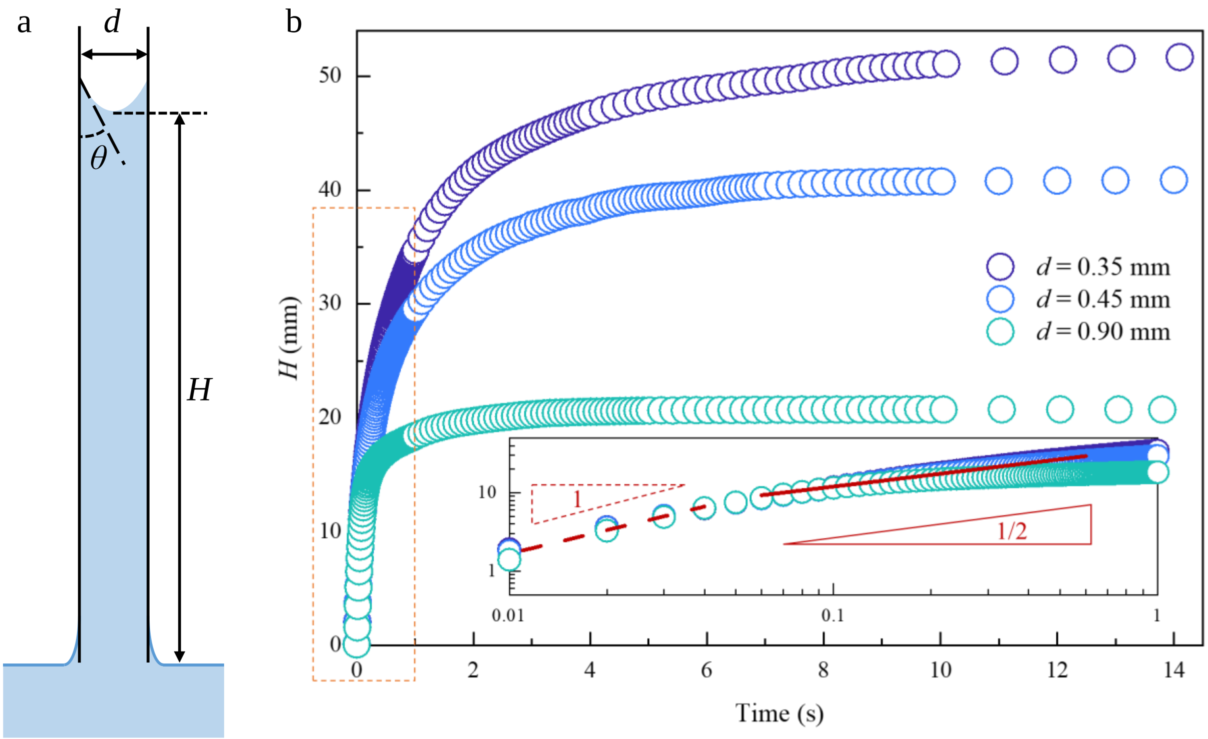

Figure S2 Dynamics of traditional capillary rise in a closed tube. (**a**) Schematic of capillary rise in a tube of diameter, d with contact angle on inside wall, θ, whose final rising height is $H \sim \sigma\cos\theta/d$; (**b**) Dynamics of capillary rise in tubes of different diameter, the inset indicates the scaling law of capillary rise to be 1 first initial stage and 1/2 for transition region.
